# 1392. Nontuberculous Mycobacteria Isolated from Wisconsin Residents, 2010-2018

**DOI:** 10.1093/ofid/ofab466.1584

**Published:** 2021-12-04

**Authors:** Bryan J Vonasek, Daniele Y Gusland, Kevin P Hash, Julie L Tans-Kersten, Suzanne N Gibbons-Burgener, Elizabeth A Misch

**Affiliations:** 1 University of Wisconsin School of Medicine and Public Health, Madison, Wisconsin; 2 Valley Children’s Healthcare, Madera, California; 3 Wisconsin Department of Health Services, Madison, Wisconsin; 4 Division of Pubic Health, Madison, Wisconsin

## Abstract

**Background:**

Wisconsin is one of a handful of states in which laboratory identification of nontuberculous mycobacteria (NTM) from clinical samples is reportable to public health. The aims of this study were to characterize the demographic features of Wisconsin adults with NTM, assess the relative abundance of NTM species recovered, and describe trends in NTM isolation over the study period.

**Methods:**

We conducted a retrospective cohort study of Wisconsin residents 18 years of age and older from whom NTM isolates were recovered and reported to the Wisconsin Electronic Disease Surveillance System (WEDSS) between 2010 and 2018. Isolates of *M. gordonae* were excluded. For the analysis of NTM frequency, multiple reports from the same individual were enumerated as separate isolates when non-identical or collected from different sites. Because NTM were usually reported into WEDSS without clinical data, this study couldn’t discern the clinical significance of the isolates.

**Results:**

A total of 9,032 NTM isolates from 7,722 adults were analyzed. The average annual number of reported NTM cases was 950 (21.7/100,000 adults) during 2011-2018. Table 1 shows the demographic characteristics of individuals with NTM isolates, stratified by specimen collection site and NTM species. *M. avium* complex (MAC) accounted for 75.7% of respiratory isolates. An important pathogenic NTM, *M. xenopi*, accounted for 8.9% of non-MAC respiratory isolates. As shown in Table 2, *M. chelonae,* a rapidly growing mycobacteria (RGM), was the most common species isolated from skin and soft tissue, head, ears, nose and throat, and eye specimens. MAC was the most common isolate from other tissue sites.

Table 1. Demographic characteristics of individuals with NTM isolates.

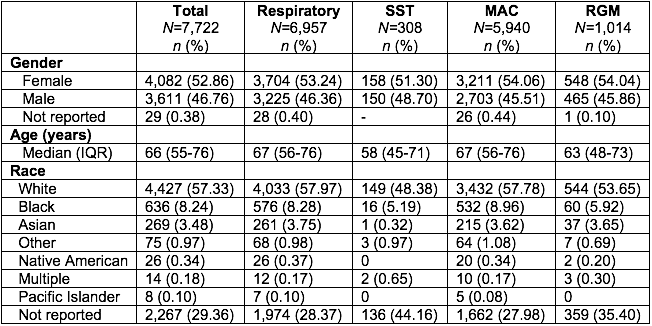

Categorization was based upon the initially recovered sample when multiple samples were obtained from a given individual. “Respiratory” samples included sputum, bronchoalveolar lavage, and tracheal aspirate specimens. IQR, interquartile range. RGM, rapidly growing mycobacteria (M. chelonae and the M. abscessus, M. chelonae-abscessus, and M. fortuitum groups). SST, skin and soft tissue.

Table 2. Most common NTM species isolated from non-respiratory sites.

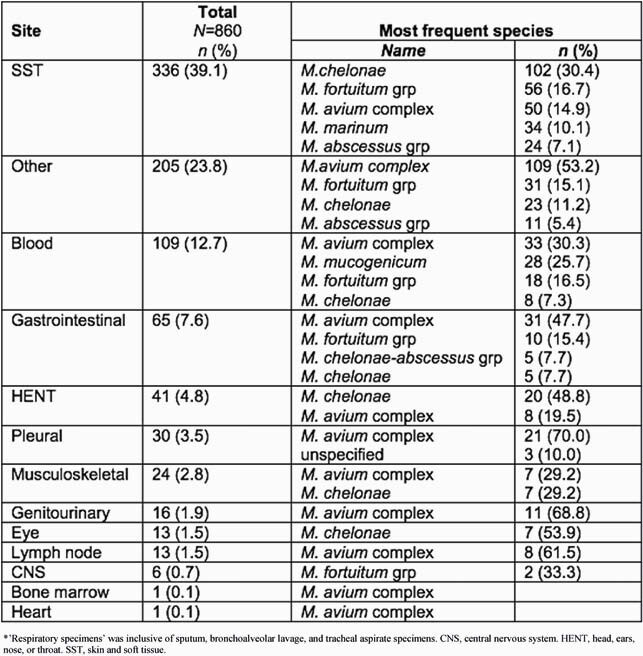

*’Respiratory specimens’ was inclusive of sputum, bronchoalveolar lavage, and tracheal aspirate specimens. CNS, central nervous system. HENT, head, ears, nose, or throat. SST, skin and soft tissue.

**Conclusion:**

Consistent with prior studies, MAC is the predominant NTM isolated from respiratory specimens in Wisconsin. RGM are important minority respiratory pathogens, and predominate as skin and soft tissue NTMs. We highlight *M. xenopi* as an important pathogen in Wisconsin compared to other parts of the United States. In contrast to recent reports of increasing incidence of NTM disease, we found a stable annual incidence of NTM isolation between 2010 and 2018.

**Disclosures:**

**All Authors**: No reported disclosures

